# The impact of HIV on presentation and outcome of bacterial sepsis and other causes of acute febrile illness in Gabon

**DOI:** 10.1007/s15010-015-0753-2

**Published:** 2015-03-11

**Authors:** Michaëla A. M. Huson, Rachel Kalkman, Sebastiaan M. Stolp, Saskia Janssen, Abraham S. Alabi, Justin O. Beyeme, Tom van der Poll, Martin P. Grobusch

**Affiliations:** 1 Division of Infectious Diseases, Center of Experimental and Molecular Medicine, Academic Medical Center, University of Amsterdam, Meibergdreef 9, Room G2-105, 1105 AZ Amsterdam, The Netherlands; 2 Centre de Recherches Médicales de Lambaréné, Albert Schweitzer Hospital, Lambaréné, Gabon; 3 Division of Infectious Diseases, Center of Tropical Medicine and Travel Medicine, Academic Medical Center, University of Amsterdam, Amsterdam, The Netherlands

**Keywords:** HIV, Sepsis, Bacteremia, Malaria, Fever, Tuberculosis

## Abstract

**Purpose:**

HIV, bacterial sepsis, malaria, and tuberculosis are important causes of disease in Africa. We aimed to determine the impact of HIV on the presentation, causes and outcome of bacterial sepsis and other acute febrile illnesses in Gabon, Central Africa.

**Methods:**

We performed a prospective observational study in new adult admissions with fever or hypothermia (≥38 or <36 °C). Blood cultures, as well as HIV and malaria testing were performed in all patients.

**Results:**

We enrolled 382 patients, including 77 (20.2 %) with HIV infection. Malaria was the most frequent diagnosis (*n* = 130, 34 %), and was associated with a more severe presentation in HIV patients. Sepsis was also common (*n* = 107, 28 %), including 29 (7.6 %) patients with culture confirmed bacterial bloodstream infection. Bacterial bloodstream infections were more frequent in HIV patients, in particular with *S. pneumoniae*. Tuberculosis was observed in 29 (7.6 %) patients, and was also more common in HIV patients. The majority of HIV patients was newly diagnosed, and only 15 (19.5 %) were using combination antiretroviral therapy.

**Conclusions:**

Our findings illustrate the impact of HIV co-infection on the burden of sepsis, malaria and tuberculosis in Gabon, as well as the need to scale up HIV counseling, testing and treatment.

**Electronic supplementary material:**

The online version of this article (doi:10.1007/s15010-015-0753-2) contains supplementary material, which is available to authorized users.

## Introduction

The HIV epidemic is a major health problem in Africa that has changed the spectrum of acute illnesses, such as sepsis and malaria, in patients presenting to the hospital [1, 2]. Sepsis is a heterogeneous condition that can be defined by the presence of clinical evidence for infection and at least two of four Systemic Inflammatory Response Syndrome (SIRS) criteria [3]. Sepsis is an important cause of morbidity and mortality worldwide, but detailed information on sepsis epidemiology in low- and middle income regions is largely unavailable. The immense burden of the HIV epidemic in Africa is likely to contribute to the number of sepsis cases in this region, as HIV patients are more likely to present with bacterial bloodstream infections (BSIs) [2]. Blood culture is an important diagnostic tool in the assessment of a septic patient, but microbiology laboratory facilities are often limited in developing regions. Therefore, gaining more insight into causative pathogens is vital to guide empirical antibiotic treatment. In Central Africa, studies on bacterial sepsis have been very limited, and no previous studies were performed in Gabon [4]. The HIV prevalence in adults in Gabon was estimated to be around 4 % in 2012 [5], and likely influences the spectrum of disease in acutely ill patients, including bacterial BSIs and sepsis. Other major causes of febrile illness in Gabon include malaria which is endemic throughout the country and is predominantly caused by *Plasmodium falciparum* [6]. The prevalence of malaria might also be influenced by the HIV epidemic. In a systematic review, malaria was identified as the third cause of morbidity in HIV patients in Africa [7], and HIV infection is associated with an increased prevalence of symptomatic malaria, particularly in patients with severe immunosuppression [1]. In addition, tuberculosis is highly prevalent in central Africa [8], and is an important cause of morbidity and mortality in Gabon, particularly in HIV-positive patients [9].

With this study, we aimed to determine the influence of HIV infection on (1) patient characteristics, including factors related to health care-seeking behavior, and main diagnoses in newly admitted patients who present with signs of SIRS, (2) the prevalence of bacterial BSI, and causative pathogens in this patient population, and (3) patient outcome.

## Materials and methods

### Setting

Patients were recruited at the Albert Schweitzer Hospital (ASH) in Lambaréné, Gabon, from March 2012 until July 2013. The ASH is a 150-bed referral hospital in the Moyen Ogooué Province and its patients originate from throughout central Gabon.

### Study design and inclusion criteria

We performed a prospective observational study. All new hospital admissions in adults (age ≥18 years) who presented with fever or hypothermia (tympanic temperature ≥38 or <36 °C), and at least one other SIRS criterion (tachycardia >90/min, respiratory rate >20/min or a white blood cell count <4e9 or >12e9 g/L) [10], were eligible for participation in the study. The study was conducted according to the principles of the Declaration of Helsinki in the current version of Seoul, 2008, and was approved by the scientific review committee of the “Centre des Recherches Medicales de Lambaréné”. Written informed consent was obtained from all patients or their guardians prior to enrollment. Aerobic and anaerobic blood cultures, as well as HIV and malaria testing, were performed for all participating patients. For HIV-positive patients CD4 counts were also determined. Patients were included by the study physician during weekdays. In addition, blood culture bottles were made available to the hospital staff to perform blood cultures at night and during the weekend.

### Clinical laboratory methods

Aerobic and anaerobic blood culture vials (Becton–Dickinson, Franklin Lakes, NJ, USA) were incubated in the automated BD Bactec 9050 system for a maximum of 5 days or until the culture became positive. Standard culture-based methods were used for species identification [API Test stripes (bioMérieux, Craponne, France) and BBL Enterotubes or BBL Oxi/Ferm Tube (Becton–Dickinson, Franklin Lakes, NJ, USA)]. Coagulase-negative staphylococci and Bacillus spp. were routinely considered contaminants. *Streptococcus viridians* were regarded contaminants as well, unless the patient had clinical signs of endocarditis or meningitis. As part of a clinical trial requirement, the microbiology laboratory at the ASH successfully participates in regular external quality assurance programs addressing species identification.

For HIV testing, a rapid test was used [Vikia HIV 1/2 (bioMérieux, Craponne, France), or Determine^TM^ HIV 1/2, (Alere, Yavne, Israel), depending on local availability]. In case of a positive reading, the result was confirmed by VIDAS HIV DUO Ultra (bioMérieux, Craponne, France) and Immunocomb HIV1&2 Bispot (Alere, Yavne, Israel). CD4 counts were done using BD FACS count (Becton–Dickinson, Franklin Lakes, NJ, USA). The Lambaréné method to analyze thick smears was applied to diagnose malaria [11].

### Case definitions

Sepsis cases were defined by clinical evidence of infection combined with fever or hypothermia (tympanic temperature ≥38 or <36 °C) and at least one other SIRS criterion. Infection diagnoses were classified on the basis of Centers for Disease Control and Prevention and International Sepsis Forum Consensus Conference definitions [12–14], adapted to the Gabonese situation where limited diagnostic tests were available (Online Resource 1: Case definitions for sites of infection in sepsis patients). Malaria cases were defined as *Plasmodium* spp. parasitemia (any parasite density) plus fever (≥38.0 °C) without another established cause of fever (blood culture negative). All patients who were treated with antituberculous drugs were classified as tuberculosis patients. In addition, for patients not (yet) on treatment, and for classification of the site of infection, we used the following definitions: Pulmonary tuberculosis was defined by the presence of at least two symptoms (chronic cough >2 weeks, night sweats, weight loss or fever) combined with a chest X-ray typical for pulmonary tuberculosis, a sputum positive smear by Ziehl–Neelsen or auramine staining. Extrapulmonary tuberculosis was identified by at least two symptoms (night sweats, weight loss or fever) combined with typical abnormalities on echography (including periportal or para-aortic lymphadenopathy, splenic microabscesses, ascites, pericardial or pleural effusion), on sight during surgical procedures or in cell compositions of specimens such as pleural or pericardial fluid, or by a positive Ziehl–Neelsen or auramine staining of an extrapulmonary site.

### Statistical methods

Categorical variables are presented as percentages and continuous variables are presented as medians with their inter quartile range (IQR). A Kolmogorov–Smirnov test was used to determine the distribution of continuous variables. We used *χ* ^2^ tests for comparisons of categorical variables, Mann–Whitney *U* tests to assess differences in non-normally distributed continuous variables, and unpaired *t* tests for normally distributed variables. For multivariate analyses of continuous variables, the Kruskal Wallis test was used for non-normally distributed variables, and one-way ANOVA for normally distributed variables. A *p* value of <0.05 was applied as level of significance in all analyses. Data analyses and creation of figures were done with SPSS statistics version 19 (IBM, Armonk, NY, USA) and GraphPad Prism (GraphPad Software, La Jolla, CA, USA).

## Results

### Patient characteristics and main diagnoses

We invited 405 eligible patients to participate in the study. Informed consent was obtained from 384 patients. Twenty-one (5.2 %) patients refused participation to the study, the main reason for refusal being unwillingness to be tested for HIV. In two patients, blood draw was technically impossible, leaving 382 patients for inclusion in our analyses. Of 382 patients, 77 (20.2 %) were HIV positive (Online Resource 2: Study flowchart). Our patient population was relatively young (median age 34 years, IQR 25–46), with a female predominance (*n* = 240, 63.8 %) (Table 1). HIV patients were slightly older than HIV-negative patients (37 years compared to 33 years, *p* = 0.04), and had a longer duration of complaints prior to presentation [4 days (IQR 1–11) compared to 3 days (IQR 2–5), *p* ≤ 0.0001]. The majority of patients came from the vicinity of the hospital (median travel time 0.5 h, IQR 0.3–1 h), although patients with HIV tended to travel farther (median travel time 0.5 h (IQR 0.5–1.6 h). HIV patients presented more frequently with respiratory symptoms, like cough (39 (50.6 %) versus 79 (25.9 %) in HIV-negative patients (*p* < 0.0001)) or shortness of breath (29 (37.7 %) and 70 (23.0 %), respectively, *p* < 0.009)), and weight loss (45 (58.4 %) versus 47 (15.4 %), respectively, *p* < 0.0001)) (Online Resource 3: Clinical symptoms and signs of patients admitted to the Albert Schweitzer hospital with acute febrile illness). The vast majority of patients were included with fever (median temperature 38.9 °C (IQR 38.6–39.0 °C); only 4 (1.0 %) patients had hypothermia on admission, and there were no differences in temperature according to HIV status. Chronic co-morbidities other than HIV infection at the time of admission were seen in 49 patients (12.8 %), the most important being hypertension (*n* = 26, 6.8 %), which was less common in HIV patients (1.3 % compared to 9.5 %, *p* = 0.02). Other co-morbidities included diabetes mellitus (*n* = 6, 1.6 %), and sickle cell disease (*n* = 4, 1.0 %).

**Table 1:** Characteristics of patients admitted to the ASH with acute febrile disease

	Total (*n* = 382)	HIV+ (*n* = 77)	HIV− (*n* = 305)	*p* value	OR (95 % CI)
Demographics					
Age (years)	34 (25–46)	37 (31–45)	33 (23–47)	**0.04**	
Male sex	142 (37.2)	22 (28.6)	120 (31.4)	0.08	0.62 (0.36–1.06)
Duration of complaints (days)	3 (2–6)	4 (1–11)	3 (2–5)	**<0.0001**	
Travel time (h)	0.5 (0.3–1.0)	0.5 (0.5–1.6)	0.5 (0.3–1.0)	**0.005**	
Diagnoses					
Malaria	130 (34.1)	14 (18.2)	116 (38.0)	**0.001**	**0.36 (0.19–0.68)**
Malaria parasites/μL^a^	7200 (1560–24000)	54000 (21990–127200)	5040 (1425–18000)	**0.0003**	
Sepsis	107 (28.1)^b^	26 (33.8)	81 (26.6)	0.21	1.41 (0.82–2.41)
Blood culture positive sepsis	30 (7.9)^b^	12 (15.6)	18 (5.9)	**0.005**	**2.95 (1.36–6.44)**
Abdominal infection^b^	41 (38.3)	5 (19.2)	36 (44.4)	**0.02**	**0.30 (0.10–0.87)**
Pneumonia	22 (20.6)	9 (34.6)	13 (16.0)	**0.02**	**3.45 (1.23–9.66)**
Skin or soft tissue infection	26 (24.3)	8 (30.8)	18 (22.2)	0.38	1.56 (0.58–4.16)
Urinary tract infection	11 (10.3)	4 (15.4)	7 (8.6)	0.32	1.92 (0.51–7.18)
Primary bacteremia	7 (6.5)	0 (0.0)	7 (8.6)	0.19	0.17 (0.01–2.97)^c^
Tuberculosis	29 (7.6)^d^	17 (22.1)	12 (3.9)	**<0.0001**	**6.92 (3.14–15.24)**
Pulmonary tuberculosis	22 (75.9)^d^	13 (76.5)	9 (75.0)	0.92	1.08 (0.19–6.06)
Ziehl–Neelsen or auramine confirmed	13 (44.8)	6 (35.3)	7 (58.3)	0.22	0.39 (0.09–1.78)

For categorical variables, the absolute number is given with the percentage, and for continuous variables medians are given with their interquartile range. We used *χ* ^2^ tests for comparisons of categorical variables, Mann–Whitney *U* tests to assess differences for non-normally distributed continuous variables, and unpaired *t* tests for normally distributed variables

All *p *values and odds ratios that breached statistical significance (*p * < 0.05) are depicted in bold

*OR* odds ratio, *CI* confidence interval

^a^All malaria cases were caused by *Plasmodium falciparum*

^b^Abdominal infections include gastroenteritis, biliary tract infections, peritonitis, appendicitis, endometritis, and intra-abdominal abscesses

^c^0.5 was added to each value to allow for calculation of an odds ratio

^d^Including two cases with both blood culture positive sepsis and pulmonary tuberculosis in one HIV-positive and one HIV-negative patient

Malaria was the most frequent diagnosis with 130 (34.1 %) cases (Table 1). HIV patients were less likely to present with malaria (18.2 % compared to 38.0 %, *p* < 0.0001), but when they had malaria, their parasitemia was higher (54000/μL compared to 5040/μL, *p* = 0.001). Sepsis was diagnosed in 107 (28.1 %) patients. Pneumonia was more frequent in HIV-positive patients (9 (34.4 %) compared to 13 (16.0 %) in HIV-negative patients, *p* = 0.02), while the reverse was true for abdominal infections (5 (19.2 %) versus 36 (44.4 %), respectively, *p* = 0.02). Other sites of infection were less frequent and were not differentially distributed according to HIV status. Positive blood cultures were present in 29 (7.6 %) patients and were more often found in HIV-positive patients (15.6 % compared to 5.9 %, OR 2.95; 95 % CI 1.36–6.44). We found no cases of bacterial BSI combined with *Plasmodium* parasitemia, but in two cases, one HIV-positive and one HIV-negative patient, a bacterial BSI was combined with pulmonary tuberculosis. As expected, tuberculosis was more frequently diagnosed in HIV-positive patients [22.1 versus 3.9 % in HIV-negative patients (OR 6.92; 95 % CI 3.14–15.24)].

The majority of HIV patients in our cohort were newly diagnosed with HIV during participation in the study (*n* = 42, 54.5 %) (Table 2). Among patients whose HIV status was already known, 21 (27.3 %) had a history of treatment with combination antiretroviral treatment (cART). However, six patients abandoned treatment, so at the time of admission only 15 patients (19.5 %) were on cART. CD4 counts were available for 68 (88.3 %) patients, and 47 (69.1 %) of these had advanced disease with CD4 counts below 350 cells/mm^3^. Median CD4 count was 168 cells/mm^3^ (IQR 61–738 cells/mm^3^) and did not differ between patients with or without cART, nor between various diagnostic categories (Table 2). The patients who abandoned treatment suffered from progressive disease with a median CD4 count of 48 cells/mm^3^ (IQR 39–231 cells/mm^3^).

**Table 2:** Characteristics of HIV-positive patients admitted with acute febrile disease

	HIV-positive patients (*n* = 77)	*p* value
HIV status known prior to admission	35 (45.5)	–
Patients with a history of cART	21 (27.3)	–
Patients on cART	15 (19.5)	–
CD4 count (*n* = 68)	168 (61–438)	–
CD4 count in patients on cART (*n* = 14)	273 (41–578)	0.33
CD4 count in patients with no previous cART (*n* = 50)	168 (73–458)	
CD4 count in patients who abandoned cART (*n* = 5)	48 (39–231)	
CD4 count in malaria cases (*n* = 13)	377 (121–730)	0.26
CD4 count in sepsis cases (*n* = 22)	168 (95–398)	
CD4 count in culture confirmed BSI cases (*n* = 12)	150 (67–198)	
CD4 count in tuberculosis cases (*n* = 16)	97 (46–158)	

For categorical variables, the absolute number is given with the percentage, and for continuous variables medians are given with their interquartile range. Multivariate analysis was done using the Kruskal–Wallis test

*cART* combination antiretroviral therapy, *BSI* bloodstream infection

Data on health care-seeking behavior were available for 379 (99.2 %) patients (Table 3). The majority (*n* = 295, 77.8 %) had taken some form of medication for their current illness prior to presenting at the hospital, the most common being analgesics (*n* = 234, 79.4 %), antimalarials (*n* = 79, 26.7 %), and antibiotics (57, 19.3 %) (Table 3). Analgesics were more frequently used by patients without HIV infection, while antibiotics were taken more often by HIV-infected patients. The main sources of medication prior to hospitalization were pharmacies (*n* = 93, 32.7 %), hospitals (*n* = 84, 29.6 %), and dispensaries (*n* = 36, 12.7 %). Patients with HIV infection had more often visited a hospital prior to presentation (OR 2.88; 95 % CI 1.56–5.29), while HIV-negative patients made more use of informal sources of medication, including friends and local shops (OR 0.17; 95 % CI 0.04–0.75). Notably, antimalarials and antibiotics were only obtained from formal health services like pharmacies, dispensaries and hospitals.

**Table 3:** Health care-seeking behavior prior to hospitalization

	Total (*n* = 379)	HIV+ (*n* = 77)	HIV− (*n* = 302)^a^	*p* value	OR (95 % CI)
Medication for current illness^b^					
Total	295 (77.8)	56 (72.7)	242 (80.1)	0.1375	0.65 (0.36–1.15)
Analgesics	234 (79.4)	35 (62.5)	200 (82.3)	**0.0011**	**0.36 (0.19–0.68)**
Antimalarials	79 (26.7)	11 (19.6)	68 (28.0)	0.2408	0.63 (0.31–1.29)
Antibiotics	57 (19.3)	20 (35.7)	37 (15.2)	**0.0004**	**3.1 (1.6–5.9)**
Other	19 (6.4)	7 (12.5)	12 (4.9)	**0.0365**	**2.75 (1.03–7.34)**
Source of medication^c^					
Pharmacy	93 (32.7)	14 (25.9)	79 (33.9)	0.1927	0.63 (0.32–1.25)
Hospital	84 (29.6)	26 (46.4)	58 (24.9)	**0.0005**	**2.88 (1.56–5.29)**
Other hospital	55 (19.4)	18 (32.1)	37 (15.9)	**0.0033**	**2.65 (1.36–5.16)**
Study hospital	29 (10.2)	8 (14.3)	21 (9.0)	0.1644	1.85 (0.77–4.47)
Dispensary	36 (12.7)	7 (12.5)	28 (12.4)	0.8482	1.09 (0.61–3.67)
Traditional medicine	5 (1.8)	1 (1.8)	3 (1.3)	0.7462	1.46 (0.45–2.65)
Various informal	44 (15.5)	2 (3.7)	42 (18.0)	**0.0085**	**0.17 (0.04–0.75)**

For categorical variables, the absolute number is given with the percentage, and for continuous variables medians are given with their interquartile range. We used *χ* ^2^ tests for comparisons of categorical variables, Mann–Whitney *U* tests to assess differences for non-normally distributed continuous variables, and unpaired *t* tests for normally distributed variables

All *p* values and odds ratios that breached statistical significance (*p* < 0.05) are depicted in bold

*OR* odds ratio, *CI* confidence interval

^a^Information on medication use prior to hospitalization was missing in 3 cases

^b^This includes only medication taken within 7 days prior to hospitalization

^c^Information on source of medication was available for 284 patients (96.3 %). Percentages in this part of the table are calculated using the number of cases for which data were available as denominator

### Bacterial bloodstream infections

In our prospective cohort of patients included by the study physician, culture confirmed true bacterial BSIs were present in 30 patients (7.9 %). An additional 84 blood cultures were taken by hospital staff in the absence of the study physician. Ten of these cultures yielded bacterial growth with a pathogenic organism (11.9 %), bringing the total number of bacterial isolates to 40. The main isolates were *Escherichia coli* (*n* = 12, 30.0 %), *Staphylococcus aureus* (*n* = 6, 15.0 %), and *Streptococcus pneumoniae* (*n* = 5, 12.5 %) (Table 4). When we stratified the causative pathogens according to HIV status, we found that *S. pneumoniae* was exclusively present in patients with HIV co-infection and this difference was statistically significant (*p* = 0.003). For other pathogens, no statistical differences were found.

**Table 4:** Main causative pathogens of bacterial bloodstream infection and their distribution according to HIV status

	Total (*n* = 40)	HIV+ (*n* = 15)	HIV− (*n* = 23)	*p* value	OR (95 % CI)
*Escherichia coli*	12 (30.0)^a^	3 (20.0)	8 (34.8)	0.33	0.47 (0.10–2.16)
*Staphylococcus aureus*	6 (15.0)^a^	3 (20.0)	2 (8.7)	0.31	2.63 (0.38–18.00)
*Streptococcus pneumoniae*	5 (12.5)	5 (33.3)	0 (0.0)	**0.003**	**24.62 (1.24–487.5)** ^b^
*Salmonella typhi*	2 (5.0)	0 (0.0)	2 (8.7)	0.24	0.28 (0.01–6.20)^b^
Non-typhoidal salmonellae	2 (5.0)	2 (13.3)	0 (0.0)	0.09	7.58 (0.34–169.0)^b^
*Klebsiella pneumoniae*	1 (2.5)	0 (0.0)	1 (4.3)	0.41	0.48 (0.02–12.68)^b^
*Serratia marcescens*	1 (2.5)	0 (0.0)	1 (4.3)	0.41	0.48 (0.02–12.68)^b^
*Streptococcus viridans*	1 (2.5)	1 (6.7)	0 (0.0)	0.21	4.86 (0.19–127.6)^b^
*Neisseria meningitidis*	1 (2.5)	0 (0.0)	1 (4.3)	0.41	0.48 (0.02–12.68)^b^
*Bacteroides* spp.	1 (2.5)	0 (0.0)	1 (4.3)	0.41	0.48 (0.02–12.68)^b^
B-Hemolytic streptococci^c^	8 (25.0)	1 (8.3)	7 (30.4)	0.08	0.16 (0.02–1.50)

For all variables, the absolute number is given with the percentage. *χ* ^2^ tests were used for comparisons between HIV-positive and HIV-negative cases

All *p* values and odds ratios that breached statistical significance (*p* < 0.05) are depicted in bold

*OR* odds ratio, *CI* confidence interval

^a^Including one case with an unknown HIV status

^b^0.5 was added to each value to allow for calculation of an odds ratio

^c^Including group B (3), group C (3), and group D (2) streptococci. The single HIV-positive patient in this group was infected with a group C streptococci

### Patient outcome

Median length of stay (LOS) in our cohort was 4 days (IQR 2–6) (Table 5). Malaria patients had the shortest duration of stay (3 days, IQR 3–4), sepsis patients stayed longer (5 days, IQR 3–8), and patients with tuberculosis had the longest LOS (13 days, IQR 9–14). The long duration of hospitalization in tuberculosis patients was due to the policy that patients should take the first ten days of antituberculous medication under supervision in the hospital. Overall mortality in our cohort was low (*n* = 16, 4.2 %), as patients with malaria had only 0.8 % mortality (*n* = 1). Patients with culture confirmed bacterial sepsis had the highest mortality of 17.2 % (*n* = 5). Patients with HIV co-infection had a longer LOS (median 5 days (IQR 3–9), compared to 4 days (IQR 3–5), *p* = <0.0001), and mortality was higher (13 % compared to 2 %). When stratified according to the main diagnostic categories, LOS was significantly longer for HIV patients with malaria, but not for sepsis or tuberculosis. Mortality was higher in HIV patients in all diagnostic categories, but the difference did not reach statistical significance in the subgroup of culture proven sepsis patients and tuberculosis patients, most likely due to the small sample size of these groups.

**Table 5:** Patient outcome

	Total (*n* = 382)	HIV+ (*n* = 77)	HIV− (*n* = 305)	*p* value	OR (95 % CI)
Length of stay (days)					
Total	4 (2–6)	5 (3–9)	4 (3–5)	**<0.0001**	
Malaria	3 (3–4)	4 (4–6)	3 (3–4)	**0.001**	
Sepsis	5 (3–8)	5 (3–10)	5 (3–7)	0.63	
Culture proven sepsis	5 (3–9)	8 (3–12)	4 (3–7)	0.10	
Tuberculosis	13 (9–14)	11 (5–15)	13 (10–14)	0.84	
In hospital mortality					
Total	16 (4.2)	10 (13.0)	6 (2.0)	**<0.0001**	**7.43 (2.61–21.18)**
Malaria	1 (0.8)	1 (7.1)	0 (0.0)	**0.005**	**24.10 (0.94–620.2)** ^**a**^
Sepsis	7 (6.5)	4 (15.4)	3 (3.7)	**0.04**	**4.73 (0.98–22.73)**
Culture proven BSI	5 (17.2)	3 (25.0)	2 (11.8)	0.35	2.50 (0.35–17.95)
Tuberculosis	3 (11.1)	3 (18.8)	0 (0.0)	0.13	5.96 (0.28–128.0)^a^

For categorical variables, the absolute number is given with the percentage, and for continuous variables medians are given with their interquartile range. We used *χ* ^2^ tests for comparisons of categorical variables, Mann–Whitney *U* tests to assess differences for non-normally distributed continuous variables, and unpaired *t* tests for normally distributed variables

All *p* values and odds ratios that breached statistical significance (*p* < 0.05) are depicted in bold

*OR* odds ratio, *CI* confidence interval, *BSI* bloodstream infection

^a^0.5 was added to each value to allow for calculation of an odds ratio

## Discussion

We demonstrated that HIV co-infection has a major impact on the causes and outcome of acute febrile illness requiring hospitalization in Gabon. In malaria patients, HIV co-infection was associated with higher parasitemia, longer LOS in hospital, and higher mortality. In accordance, previous studies found increased severity of disease and impaired response to treatment in HIV-positive malaria patients [1]. This could be due to defects in the innate immune response to malaria, as natural killer, natural killer T, and γδ T-cells, isolated from the peripheral blood of HIV patients, were recently shown to be less responsive to a challenge with *P. falciparum* parasites [15]. The prevalence of malaria in our cohort was high (34.1 %), and our figures are even likely to present an underestimation as use of antimalarial drugs prior to hospitalization was common. HIV infection was previously described as a risk factor for symptomatic malaria [1], so the HIV epidemic in Gabon may drive an increase in adult malaria cases. As malaria infection was even more frequent in HIV-negative patients (38 %), other factors are likely adding to the burden of malaria. Recent evidence indicates that malaria is an underrecognized driver of adult mortality in Africa [16], and one possibility is that the successful roll-out of malaria intervention strategies targeted at children and pregnant women has shifted the burden of disease to non-pregnant adults [17, 18].

Sepsis was an important reason for admission in HIV-positive patients (33.8 %), and HIV-negative patients (26.6 %). In line with previous studies, HIV co-infection was associated with an increased prevalence of bacterial BSI in patients with sepsis, and enhanced sepsis-related mortality [2]. The relatively low overall prevalence of bacterial BSI in this cohort compared to that reported in a systematic review for African countries (7.6 % compared to 13.5 %) may be related to the fact that our population was relatively young and had few chronic co-morbidities. In accordance with previous studies [2, 4], we found *E. coli, S. aureus* and *S. pneumoniae* to be the main isolates in patients with BSI, and our study confirms an increased risk for *S. pneumoniae* BSI in HIV-positive patients, a pathogen that is known to frequently cause invasive disease in HIV patients [4, 19]. Bloodstream infection with non-typhoidal *Salmonella,* a pathogen also known to be more common in patients with HIV co-infection [2, 20], was exclusively found in HIV-positive patients, but the number of cases was too small to demonstrate a significant difference with HIV-negative patients. Pathogenic mechanisms that may be related to increased prevalence and severity of bacterial sepsis were recently reviewed, and include defects in both innate and adaptive immunity [21].

Tuberculosis is an important cause of morbidity and mortality in HIV patients [22]. In accordance, we observed a higher prevalence of tuberculosis in HIV-positive patients (22.1 % compared to 3.9 %). Key factors for the increased susceptibility of HIV patients to develop tuberculosis include general as well as *Mycobacterium tuberculosis* specific CD4 T cell depletion [22]. In comparison to a previous retrospective survey of tuberculosis in the same hospital, which registered an annual number of around 120 tuberculosis cases [9], the number of tuberculosis patients in this study is low (*n* = 29). A possible explanation for this difference is that the majority of hospitalized tuberculosis patients do not present with acute febrile illness, but rather are admitted with chronic complaints. The higher rate of HIV co-infection in tuberculosis patients in our cohort (58.6 %), compared to 28.5 % in the previous study [9], suggests that HIV patients with tuberculosis are more prone to present with an acute sepsis-like illness.

Non-prescription antibiotic use is common in Africa and is a driver of antimicrobial resistance [23]. In our cohort, 19.3 % used antibiotics prior to hospitalization, but the majority of patients obtained their drugs from a previous visit to the hospital, indicating that the use of over-the-counter antibiotics is limited in Gabon. In HIV patients, the use of antibiotics for their current illness prior to hospitalization was higher, which was most likely related to increased severity and longer duration of illness. These patients were also more likely to have visited a hospital for their complaints prior to their hospitalization. Antimalarial drugs were more commonly used prior to hospitalization (26.7 % of cases with no difference according to HIV status) and were mostly bought in the pharmacy. These figures are low in comparison to other African countries, such as Nigeria, where a survey found 100 % of antibiotics used were obtained without prescription [23]. The use of traditional medicine was previously reported to be very common in Gabonese patients with tuberculosis [24], and is also frequently observed in HIV patients in other African countries [25, 26]. However, in our study the use of traditional medicine was limited to 1.8 % of patients, with no differences according to HIV status. One explanation for the discrepancy is that traditional medicine is mostly used in the case of chronic illness, whereas our patients generally presented to the hospital with acute illness. In addition, patients may be reluctant to discuss the use of traditional medicine in a formal health care setting like the hospital.

Our data illustrate the burden of HIV infection in this region and gaps in HIV testing and treatment. The majority of HIV patients (69.1 %) presented with advanced disease with CD4 counts below 350 cells/mm^3^, the current World Health Organization threshold for prioritizing the start of cART [27]. However, only 19.5 % of HIV patients were using treatment at the time of admission. The need for increased availability of HIV counseling and testing is illustrated by the fact that more than half (54.5 %) of the HIV patients in our cohort were newly diagnosed. In addition, the finding that 20.2 % of patients in our cohort were HIV positive illustrates that implementation of routine counseling and testing for hospitalized patients would be beneficial. In other African countries, provider-initiated testing and counseling proved to be very useful in increasing the number of patients tested and in reaching patients who perceived themselves to be at low risk [28, 29]. Although patient acceptance was variable in other settings [28], our study demonstrates a high level of acceptance for HIV testing among Gabonese patients. However, challenges exist in linking newly diagnosed patients to care and retaining them in care. Previous studies in Gabon and other settings demonstrated a high level of loss to follow-up in HIV patients and we also found that a significant proportion of patients who previously used treatment abandoned cART (*n* = 6, 28.6 %) [29].

There are some limitations of this study that need to be mentioned. First, due to limited availability of diagnostic tools, we were unable to provide a conclusive diagnosis for the entire cohort. Possible diagnoses in febrile patients who did not meet our criteria for malaria, sepsis or tuberculosis include viral illnesses, such as dengue or chikungunya, both of which have been previously reported in Gabon [30] as well as malaria episodes masked by previous treatment. In addition, as the study was not primarily designed to investigate tuberculosis, the indication for tuberculosis diagnostic tests was left to the treating physician. Tuberculosis was principally diagnosed by Ziehl Neelsen staining of sputum, or on clinical grounds, as alternative diagnostic means such as culture or PCR were not available. This may have resulted in an underestimation of the number of tuberculosis cases in this cohort. Third, we performed a hospital-based study, so our findings may not be representative for causes of morbidity and mortality in the community. Although the Albert Schweitzer Hospital is a regional referral hospital, the vast majority of patients came from the vicinity of the hospital, which suggests that patients in remote areas with limited infrastructure, particularly those with more severe disease, may not be able to reach the hospital.

## Conclusion

In African countries like Gabon, the HIV epidemic has a major influence on the prevalence, severity and outcome of acute febrile illness due to sepsis, malaria or tuberculosis. This study is the first to systematically investigate bacterial BSIs and sepsis in Gabon, and illustrates the importance of microbiology laboratory facilities, in particular for HIV patients who present more frequently with bacterial BSI. In addition, we identified a large gap between the number of HIV patients in need of cART, and those who know their HIV status and receive proper treatment or care, representing an important challenge in the management of HIV in this region.

## Electronic supplementary material

Below is the link to the electronic supplementary material.

Supplementary material 1 (PDF 264 kb)

Supplementary material 2 (PDF 254 kb)

Supplementary material 3 (PDF 358 kb)
