# Supplementary material for: The impact of HIV on presentation and outcome of bacterial sepsis and other causes of acute febrile illness in Gabon
Source: Infection. 2015 Mar 11;43(4):443–51. doi: 10.1007/s15010-015-0753-2 (PMC4521089; doi:10.1007/s15010-015-0753-2)
Supplement: Supplementary file 1 — Supplementary material 1 (PDF 264 kb) [file 15010_2015_753_MOESM1_ESM.pdf]

Huson MA*, Kalkman R, Stolp SM, Janssen S, Alabi AS, Beyeme JO, van der Poll T, Grobusch MP. The impact of HIV on presentation and outcome of bacterial sepsis and other causes of acute febrile illness in Gabon. *Infection*

*Correspondence: Academic Medical Center, Meibergdreef 9, room G2-105, 1105 AZ Amsterdam, The Netherlands, m.a.huson@amc.uva.nl

Online Resource 1: Case definitions for sepsis

Primary bacteremia

Primary bacteremia was defined as a positive blood culture in the absence of localizing symptoms or signs.

Pulmonary infections

Pulmonary infections were characterized by the presence of at least two symptoms of pneumonia (cough, production of purulent sputum, hypoxia (>90% O2 saturation) or rales on physical examination) combined with an infiltrate on the chest X-ray or a positive blood culture with a likely respiratory pathogen.

Abdominal infections

Gastroenteritis was defined as acute diarrhoea (>12h, minimal 5/day or 500ml or watery stools) without a clear non-infectious cause.

Biliary tract infections were identified by at least two symptoms (pain in the right upper abdomen, jaundice or colicky pain) combined with micro-organisms in the biliary tract or blood culture.

Other intra-abdominal infections, such as peritonitis, appendicitis, endometritis and intra-abdominal abcesses were confirmed by echographic imaging or surgery.

Urinary tract infections

Urinary tract infections were identified by the presence of at least two symptoms (urgency, dysuria, pyuria, hematuria, suprapubic pain or flank pain) combined with a positive blood or urine culture for

a likely urinary pathogen, a positive nitrite test on a urine stick, or a positive gram stain of a urine sediment.

Skin, soft tissue and joint infections

Skin, soft tissue and joint infections were characterized by at least 2 of the following local symptoms: pain or tenderness, swelling, redness, warmth, purulent discharge, pustules or blisters.

Meningitis

Meningitis was defined as at least two symptoms (headache, nuchal rigidity, meningism, loss of function of cranial nerves, change in mental status, or petechiae), combined with a positive blood culture, or evidence in the liquor of an infection, including a positive liquor culture, micro-organism in gram stain, increased leukocyte number (>2000/microL), increased protein (220 mg/dL), or decreased glucose (<34 mg/dL (1.9 mmol/L) in liquor.

Endocarditis

Endocarditis was defined as a positive blood culture with an organism typical for endocarditis (*Streptococcus viridans, Streptococcus bovis,* HACEK group (*Haemophilus* spp., *Actinobacillus actinomycetemcomitans, Cardiobacterium hominis, Eikenella* spp.*, Kingella kingae*), *Staphylococcus aureus*, *Enterococcus* spp., *Coxiella burnetii*) combined with a cardiac murmur or echographic evidence for cardiac involvement, in the absence of an alternative diagnosis and without resolution of manifestations of endocarditis with antibiotic therapy for four days or less.
