# Supplementary material for: The impact of HIV on presentation and outcome of bacterial sepsis and other causes of acute febrile illness in Gabon
Source: Infection. 2015 Mar 11;43(4):443–51. doi: 10.1007/s15010-015-0753-2 (PMC4521089; doi:10.1007/s15010-015-0753-2)

Huson MA*, Kalkman R, Stolp SM, Janssen S, Alabi AS, Beyeme JO, van der Poll T, Grobusch MP. The impact of HIV on presentation and outcome of bacterial sepsis and other causes of acute febrile illness in Gabon. *Infection*

*Correspondence: Academic Medical Center, Meibergdreef 9, room G2-105, 1105 AZ Amsterdam, The Netherlands, m.a.huson@amc.uva.nl

Online Resource 2: Study flowchart

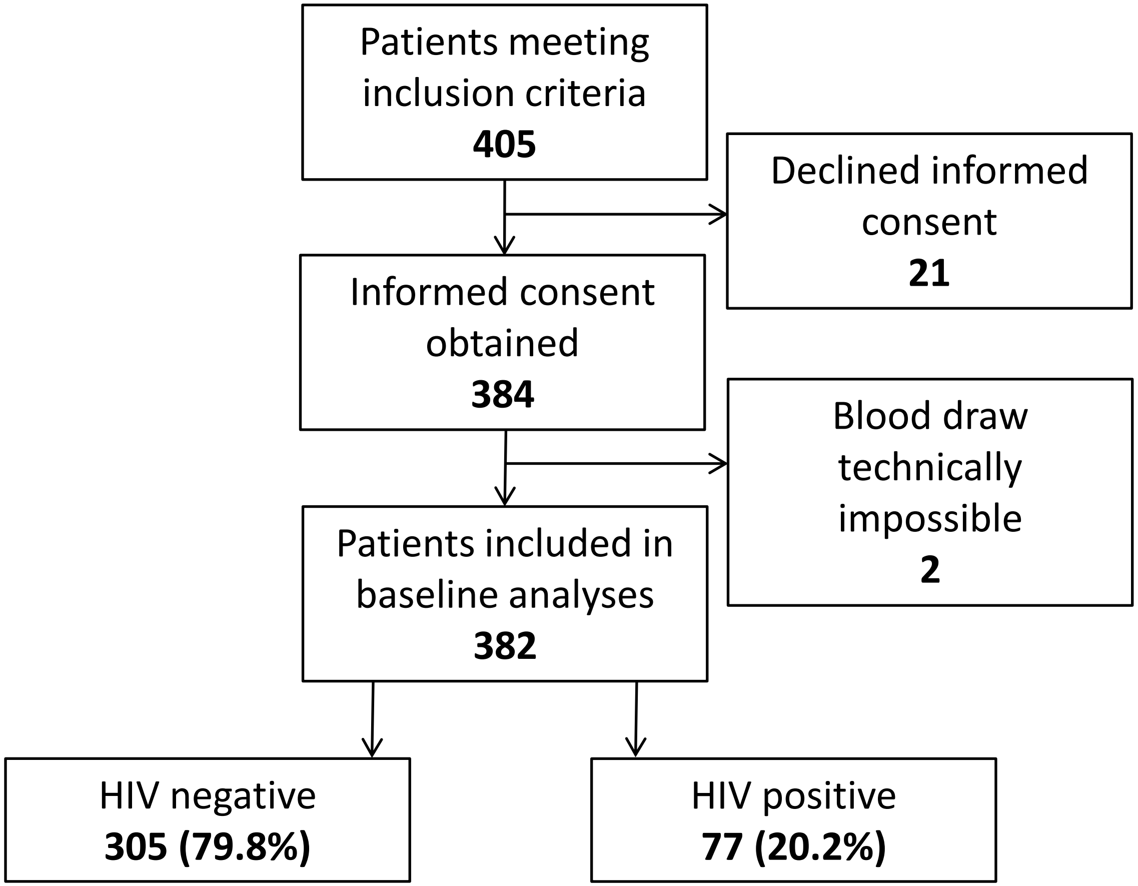

Supplement: Supplementary file 2 — Supplementary material 2 (PDF 254 kb) [file 15010_2015_753_MOESM2_ESM.pdf]
