# Supplementary material for: The impact of HIV on presentation and outcome of bacterial sepsis and other causes of acute febrile illness in Gabon
Source: Infection. 2015 Mar 11;43(4):443–51. doi: 10.1007/s15010-015-0753-2 (PMC4521089; doi:10.1007/s15010-015-0753-2)
Supplement: Supplementary file 3 — Supplementary material 3 (PDF 358 kb) [file 15010_2015_753_MOESM3_ESM.pdf]

Huson MA*, Kalkman R, Stolp SM, Janssen S, Alabi AS, Beyeme JO, van der Poll T, Grobusch MP. The impact of HIV on presentation and outcome of bacterial sepsis and other causes of acute febrile illness in Gabon. *Infection*

*Correspondence: Academic Medical Center, Meibergdreef 9, room G2-105, 1105 AZ Amsterdam, The Netherlands, m.a.huson@amc.uva.nl

Online Resource 3: Clinical symptoms and signs of patients admitted to the Albert Schweitzer hospital with acute febrile illness

	**Total n=382**	**HIV+ n=77**	**HIV- n=305**	**p-value**	**OR (95% CI)**
**Clinical symptoms**					
Headache	230 (60.2)	40 (51.9)	190 (62.3)	0.09	0.65 (0.40-1.08)
Vomiting	153 (40.1)	28 (36.4)	125 (41.0)	0.46	0.82 (0.49-1.38)
Abdominal pain	149 (39.0)	28 (36.4)	121 (39.7)	0.59	0.87 (0.52-1.46)
Cough	118 (30.9)	39 (50.6)	79 (25.9)	**< 0.0001**	**2.94 (1.75-4.91)**
Shortness of breath	99 (25.9)	29 (37.7)	70 (23.0)	**0.009**	**2.03 (1.19-3.46)**
Diarrhea	96 (25.1)	22 (28.6)	74 (24.3)	0.44	1.25 (0.71-2.19)
Weight loss	92 (24.1)	45 (58.4)	47 (15.4)	**< 0.0001**	**7.72 (4.46-13.38)**
Joint pain	87 (22.8)	7 (9.1)	80 (26.2)	**0.001**	**0.28 (0.12-0.64)**
Urinary tract symptoms	51 (13.4)	9 (11.7)	42 (13.8)	0.63	0.83 (0.38-1.79)
Night sweats	22 (5.8)	11 (14.3)	11 (3.6)	0.91	1.04 (0.51-2.12)
Altered mental status	15 (3.9)	2 (2.6)	13 (4.3)	0.50	0.60 (0.13-2.71)
Reproductive tract symptoms	14 (3.7)	1 (1.3)	13 (4.3)	0.22	0.30 (0.04-2.30)
Skin symptoms	9 (2.4)	3 (3.9)	6 (2.0)	0.32	2.02 (0.49-8.27)
**Clinical signs**					
Temperature	38.9 (38.3-39.5)	38.6 (38.3-39.4)	39.0 (38.3-39.5)	0.22	
Heart rate	104.5 (92-115)	110 (101-118)	103 (92-113)	**0.001**	
Respiratory rate	28 (24-32.5)	30 (24-36)	28 (24-32)	0.35	
Hemoglobin (g/dl)	11.4 (9.8-13)	9.7 (8.0-11.2)	11.8 (10.2-13.2)	**< 0.0001**	
Leukocyte count (cells/mm3)	6.95 (4.275-10.6)	6.8 (4.6-10.6)	7 (4.25-10.55)	0.91	
Platelets (109/L	158 (107-227.5)	189 (135.5-292.5)	149.5 (101-216)	**0.0008**	

For categorical variables the absolute number is given with the percentage, and for continuous variables medians are given with their interquartile range. We used χ^2 tests for comparisons of categorical variables, Mann Whitney U tests to assess differences for non-normally distributed continuous variables, and unpaired t-tests for normally distributed variables.
Abbreviations: OR: Odds ratio, CI: confidence interval
